# Supplementary material for: Challenges with Assessing and Treating Pain in Research Primates: A Focused Survey and Literature Review
Source: Animals (Basel). 2022 Sep 5;12(17):2304. doi: 10.3390/ani12172304 (PMC9455027; doi:10.3390/ani12172304)
Supplement: Supplementary file 1 [file animals-12-02304-s001.zip › animals-1879767-supplementary.pdf]

animals

Supplementary material

Challenges with Assessing and Treating Pain in Research Primates: A Focused Survey and Literature Review

Emilie A. Paterson [1] and Patricia V. Turner [1,2,*]

[1] Department of Pathobiology, University of Guelph, Guelph, ON N1G 0C4, Canada
[2] Global Animal Welfare and Training, Charles River, Wilmington, MA 01887, USA
* Correspondence: pvturner@uoguelph.ca

Citation: Paterson, E.A.; Turner, P.V. Challenges with Assessing and Treating Pain in Research Primates: A Focused Survey and Literature Review. *Animals* **2022**, *12*, 2304. https://doi.org/10.3390/ani12172304

Academic Editor: Garikoitz Azkona

Received: 7 August 2022
Accepted: 27 August 2022
Published: 5 September 2022

Publisher's Note: MDPI stays neutral with regard to jurisdictional claims in published maps and institutional affiliations.

Copyright: © 2022 by the authors. Licensee MDPI, Basel, Switzerland. This article is an open access article distributed under the terms and conditions of the Creative Commons Attribution (CC BY) license (https://creativecommons.org/licenses/by/4.0/).

The use of analgesics in research primates questionnaire.

1. What country do you live and work in?

2. Please select the range below that includes your age.
   - < 25
   - 25-35
   - 35-45
   - 45-55
   - 55-65
   - > 65

3. Please indicate your gender
   - Male
   - Female

4. Please indicate the type of institution that you work in.

5. Please indicate your primary job function (e.g., clinical veterinarian, researcher, toxicologist, administrator, etc).

6. Do you work with living primates in your primary job function?
   - Yes
   - No

7. If you answered yes to question 6 please select the species that you work with from the list below (check all that apply):
   - Macaques
   - Squirrel monkeys
   - Owl monkeys
   - Baboons
   - African green monkeys
   - Sooty mangabeys
   - Chimpanzees
   - Other, please specify :

8. Does your animal ethics committee have a formal experimental pain assessment policy for research primates? (If no, please skip to question 10).
   - Yes
   - No

9. If you answered 'yes' to Question 8, please indicate whether the SOP is specific to primate pain assessment (including signs to evaluate) or whether it is generic and applies to all laboratory species housed at the site. (please specify).

10. Please indicate which of the following tools are used for pain assessment in research primates at your facility, if any. (Note: more than 1 response is permitted).

Animals can't be closely observed because of housing methods
Facial grimace scoring
Indirect animal assessment (e.g., video camera)
Bruxism (teeth grinding)
Interaction with conspecifics
Change in respiration patterns
Lameness
Posture
Disuse or guarding of body part
Food consumption
Direct animal assessment (e.g., cage side)
General activity level
None of the above
Other, please specify:

11. Who is responsible for making pain assessments in research primates at your facility? (check all boxes below for whom this is applicable)

Veterinarian
Veterinary technician
Animal care personnel
Principal investigators
Students
Other research staff
Other, please specify:

12. Are animals monitored regularly after treatment to determine whether analgesia is effective?

Yes
No
Sometimes
Other, please specify:

13. How often are unplanned top-ups in analgesic medication provided to research primates at your facility to manage pain?

Never
Rarely
Sometimes
Often
Not applicable, our facility does not conduct any surgical or other study procedures requiring

14. I would recommend pain assessment and medication (if needed) routinely for the following procedures (please check all that apply):

Cranial cap cleaning and maintenance
Traumatic peripheral blood collection (requiring 3 or more attempts)
Subcutaneous RFID implant injection
Dental prophylaxis
Cage/housing/enclosure-related laceration
Finger amputation
Routine parturition with no complications
Lameness or paresis secondary to injury in a research primate on a GLP study
Intracranial guide tube placement
None of the above
We are unable to provide analgesia off protocol for research primates at our facility

15. Please indicate which of the following pharmacologic and nonpharmacologic methods are used, at times, at your facility to alleviate research primate pain.

Nonsteroidal anti-inflammatory drugs, e.g., meloxicam
Opioids, e.g., buprenorphine
Topical or local analgesic/anesthetic agents
Supplemental foods
Soft bedding or additional substrate
Alternate caging
Acupuncture
Laser therapy
Massage therapy
Hydrotherapy
Supplemental heat
None of the above
Other, please specify:

16. Please list specific nonsteroidal anti-inflammatory drugs used for treatment of pain in research primates at your facility, if any (please be specific and list all, including short and long term formulations).

17. Please list specific opioid drugs used for treatment of pain in research primates at your facility, if any (please be specific and list all, including short and long term formulations).

18. Please list specific local or topical analgesic/anesthetic drugs used for treatment of pain in research primates at your facility, if any (please be specific and list all, including short and long term formulations).

19. Please indicate your level of confidence in how well you recognize and manage pain in research primates.

Not very confident
Slightly unconfident

Neutral
Somewhat confident
Highly confident

20. Please indicate your level of confidence in how well research personnel at your facility recognize and manage pain in research primates.

Not very confident
Slightly unconfident
Neutral
Somewhat confident
Highly confident

21. Do you have any other comments to make regarding assessment of pain, methods used to alleviate pain or evaluation of analgesic efficacy in research primates?
